# 2-Amino-5-chloro­pyridine–benzoic acid (1/1)

**DOI:** 10.1107/S1600536810004447

**Published:** 2010-02-10

**Authors:** Madhukar Hemamalini, Hoong-Kun Fun

**Affiliations:** a X-ray Crystallography Unit, School of Physics, Universiti Sains Malaysia, 11800 USM, Penang, Malaysia

## Abstract

In the title compound, C_5_H_5_ClN_2_·C_7_H_6_O_2_, the carboxyl group of the benzoic acid mol­ecule is twisted away from the attached ring by 14.22 (7)°. In the crystal, the 2-amino-5-chloro­pyridine mol­ecules inter­act with the carboxyl groups of benzoic acid mol­ecules through N—H⋯O and O—H⋯N hydrogen bonds, forming cyclic *R*_2_^2^(8) hydrogen-bonded motifs, and linking the mol­ecules into chains parallel to the [001] direction. Neighbouring 2-amino-5-chloro­pyridine mol­ecules are also centrosymmetrically paired through C—H⋯Cl hydrogen bonds, forming another *R*_2_^2^(8) motif. The crystal structure is further stabilized by weak C—H⋯O hydrogen bonds.

## Related literature

For background to the chemistry of substituted pyridines, see: Pozharski *et al.* (1997 ▶); Katritzky *et al.* (1996 ▶); For details of hydrogen bonding, see: Jeffrey & Saenger (1991 ▶); Jeffrey (1997 ▶); Scheiner (1997 ▶). For hydrogen-bond motifs, see: Bernstein *et al.* (1995 ▶); Lynch & Jones (2004 ▶). For reference bond-length data, see: Allen *et al.* (1987 ▶). For the stability of the temperature controller used in the data collection, see: Cosier & Glazer (1986 ▶).
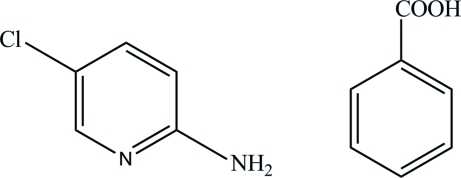


## Experimental

### 

#### Crystal data

C_5_H_5_ClN_2_·C_7_H_6_O_2_
*M* *_r_* = 250.68
Monoclinic, 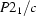

*a* = 17.6114 (19) Å
*b* = 5.3442 (6) Å
*c* = 12.4774 (13) Å
β = 100.161 (2)°
*V* = 1155.9 (2) Å^3^
*Z* = 4
Mo *K*α radiation
μ = 0.32 mm^−1^
*T* = 100 K
0.55 × 0.25 × 0.07 mm

#### Data collection

Bruker SMART APEX DUO CCD area-detector diffractometer
Absorption correction: multi-scan (*SADABS*; Bruker, 2009 ▶) *T*_min_ = 0.844, *T*_max_ = 0.979
11852 measured reflections
3331 independent reflections
2802 reflections with > 2(*I*)
*R*_int_ = 0.025

#### Refinement

*R*[*F* ^2^ > 2σ(*F* ^2^)] = 0.033
*wR*(*F* ^2^) = 0.111
*S* = 1.12
3331 reflections
198 parameters
All H-atom parameters refined
Δρ_max_ = 0.46 e Å^−3^
Δρ_min_ = −0.21 e Å^−3^

### 

Data collection: *APEX2* (Bruker, 2009 ▶); cell refinement: *SAINT* (Bruker, 2009 ▶); data reduction: *SAINT*; program(s) used to solve structure: *SHELXTL* (Sheldrick, 2008 ▶); program(s) used to refine structure: *SHELXTL*; molecular graphics: *SHELXTL*; software used to prepare material for publication: *SHELXTL* and *PLATON* (Spek, 2009 ▶).

## Supplementary Material

Crystal structure: contains datablocks global, I. DOI: 10.1107/S1600536810004447/wn2376sup1.cif

Structure factors: contains datablocks I. DOI: 10.1107/S1600536810004447/wn2376Isup2.hkl

Additional supplementary materials:  crystallographic information; 3D view; checkCIF report

## Figures and Tables

**Table 1:** Hydrogen-bond geometry (Å, °)

*D*—H⋯*A*	*D*—H	H⋯*A*	*D*⋯*A*	*D*—H⋯*A*
O2—H1*O*2⋯N1^i^	0.98 (2)	1.65 (2)	2.629 (1)	175 (2)
N2—H1*N*2⋯O1^ii^	0.88 (2)	2.04 (2)	2.898 (2)	165.4 (18)
N2—H2*N*2⋯O2^iii^	0.88 (2)	2.37 (2)	3.231 (2)	165.8 (17)
C3—H3⋯Cl1^iv^	0.99 (2)	2.82 (2)	3.780 (2)	163.4 (16)
C6—H6⋯O1^v^	0.91 (2)	2.58 (2)	3.095 (2)	116.3 (15)

Symmetry codes: (i) 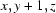; (ii) 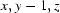; (iii) 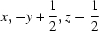; (iv) 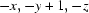; (v) 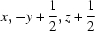.

**Fig. 1.**
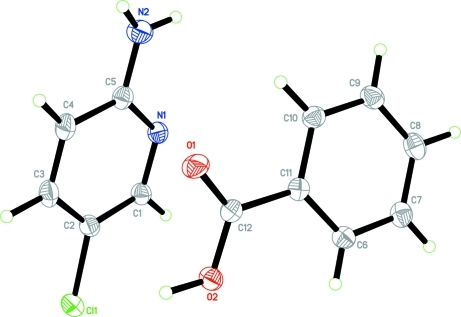
The asymmetric unit of the title compound. Displacement ellipsoids are drawn at the 50% probability level. Hydrogen atoms are shown as spheres of arbitrary radius.

**Fig. 2.**
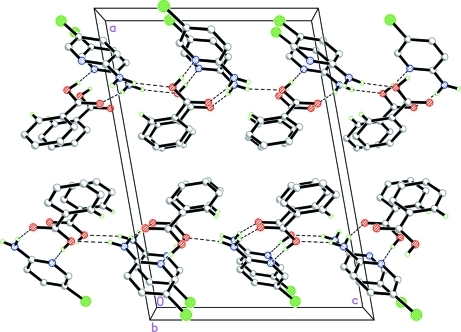
The crystal packing of the title compound, showing hydrogen-bonded (dashed lines) networks. Hydrogen atoms not involved in hydrogen bonding have been omitted.

## supplementary crystallographic information

### Comment

Pyridine and its derivatives play an important role in heterocyclic chemistry (Pozharski *et al.*, 1997; Katritzky *et al.*, 1996). Pyridine and its substituted derivatives are often involved in hydrogen-bond interactions (Jeffrey & Saenger, 1991; Jeffrey, 1997; Scheiner, 1997). The adducts of carboxylic acids with the 2-aminoheterocylic ring system form a graph-set motif *R*_2_^2^(8) (Lynch & Jones, 2004). In the present study, the hydrogen-bonding patterns in the 2-amino-5-chloropyridine benzoic acid (1/1) cocrystal, are investigated.

The asymmetric unit (Fig. 1), contains one 2-amino-5-chloropyridine molecule and one benzoic acid molecule. The 2-amino-5-chloropyridine molecule is planar, with a maximum deviation of 0.002 (1) Å for atom N1. The carboxyl group of the benzoic acid molecule is twisted away from the attached ring by 14.22 (7)° . The bond lengths (Allen *et al.*, 1987) and angles are normal.

In the crystal packing (Fig. 2), the 2-amino-5-chloropyridine molecules interact with the carboxyl group of benzoic acid molecules through N—H···O and O—H···N hydrogen bonds, forming a cyclic hydrogen-bonded motif *R*_2_^2^(8) (Bernstein *et al.*, 1995), and linking the molecules into chains parallel to the [001] direction. Neighbouring 2-amino-5-chloropyridine molecules are also centrosymmetrically paired through C—H···Cl hydrogen bonds, forming another *R*_2_^2^(8) motif. The crystal structure is further stabilized by weak C6—H6···O1 (Table 1) hydrogen bonds.

### Experimental

A hot methanol solution (20 ml) of 2-amino-5-chloropyridine (65 mg, Aldrich) and benzoic acid (61 mg, Merck) were mixed and warmed over a heating magnetic stirrer for a few minutes.  The resulting solution was allowed to cool slowly at room temperature and crystals of the title compound appeared after a few days.

### Refinement

All the H atoms were located in a difference Fourier map and allowed to refine freely [N—H = 0.88 (2) Å, O—H = 0.98 (2) Å, C—H = 0.91 (2) - 1.02 (2) Å].

### Crystal data

**Table:**

C_5_H_5_ClN_2_·C_7_H_6_O_2_	*F*(000) = 520
*M**_r_* = 250.68	*D*_x_ = 1.440 Mg m^−^^3^
Monoclinic, *P*2_1_/*c*	Mo *K*α radiation, λ = 0.71073 Å
Hall symbol: -P 2ybc	Cell parameters from 5100 reflections
*a* = 17.6114 (19) Å	θ = 2.4–30.0°
*b* = 5.3442 (6) Å	µ = 0.32 mm^−^^1^
*c* = 12.4774 (13) Å	*T* = 100 K
β = 100.161 (2)°	Plate, colourless
*V* = 1155.9 (2) Å^3^	0.55 × 0.25 × 0.07 mm
*Z* = 4	

### Data collection

**Table:**

Bruker SMART APEX DUO CCD area-detector diffractometer	3331 independent reflections
Radiation source: fine-focus sealed tube	2802 reflections with > 2(*I*)
graphite	*R*_int_ = 0.025
φ and ω scans	θ_max_ = 30.0°, θ_min_ = 1.2°
Absorption correction: multi-scan (*SADABS*; Bruker, 2009)	*h* = −24→24
*T*_min_ = 0.844, *T*_max_ = 0.979	*k* = −7→7
11852 measured reflections	*l* = −17→17

### Refinement

**Table:**

Refinement on *F*^2^	Primary atom site location: structure-invariant direct methods
Least-squares matrix: full	Secondary atom site location: difference Fourier map
*R*[*F*^2^ > 2σ(*F*^2^)] = 0.033	Hydrogen site location: inferred from neighbouring sites
*wR*(*F*^2^) = 0.111	All H-atom parameters refined
*S* = 1.12	*w* = 1/[σ^2^(*F*_o_^2^) + (0.0596*P*)^2^ + 0.2767*P*] where *P* = (*F*_o_^2^ + 2*F*_c_^2^)/3
3331 reflections	(Δ/σ)_max_ < 0.001
198 parameters	Δρ_max_ = 0.46 e Å^−^^3^
0 restraints	Δρ_min_ = −0.21 e Å^−^^3^

### Special details

**Table:**

Experimental. The crystal was placed in the cold stream of an Oxford Cryosystems Cobra open-flow nitrogen cryostat (Cosier & Glazer, 1986) operating at 100.0 (1) K.
Geometry. All esds (except the esd in the dihedral angle between two l.s. planes) are estimated using the full covariance matrix. The cell esds are taken into account individually in the estimation of esds in distances, angles and torsion angles; correlations between esds in cell parameters are only used when they are defined by crystal symmetry. An approximate (isotropic) treatment of cell esds is used for estimating esds involving l.s. planes.
Refinement. Refinement of F^2^ against ALL reflections. The weighted R-factor wR and goodness of fit S are based on F^2^, conventional R-factors R are based on F, with F set to zero for negative F^2^. The threshold expression of F^2^ > 2sigma(F^2^) is used only for calculating R-factors(gt) etc. and is not relevant to the choice of reflections for refinement. R-factors based on F^2^ are statistically about twice as large as those based on F, and R- factors based on ALL data will be even larger.

### Fractional atomic coordinates and isotropic or equivalent isotropic displacement parameters (Å^2^)

**Table:**

	*x*	*y*	*z*	*U*_iso_*/*U*_eq_	
Cl1	0.029115 (18)	0.29660 (7)	0.17435 (3)	0.02611 (11)	
N1	0.17431 (6)	−0.2004 (2)	0.08617 (9)	0.0197 (2)	
N2	0.22948 (8)	−0.2526 (3)	−0.06737 (10)	0.0270 (3)	
C1	0.12668 (7)	−0.0733 (2)	0.14079 (10)	0.0202 (2)	
C2	0.08720 (7)	0.1356 (2)	0.09858 (10)	0.0203 (2)	
C3	0.09605 (8)	0.2235 (3)	−0.00426 (11)	0.0232 (3)	
C4	0.14399 (8)	0.0960 (3)	−0.06017 (11)	0.0229 (3)	
C5	0.18308 (7)	−0.1193 (2)	−0.01300 (10)	0.0199 (2)	
O1	0.30023 (6)	0.33873 (19)	0.06701 (7)	0.0235 (2)	
O2	0.25316 (5)	0.46305 (18)	0.21358 (7)	0.0202 (2)	
C6	0.35843 (7)	0.1449 (3)	0.34708 (10)	0.0194 (2)	
C7	0.40642 (7)	−0.0304 (3)	0.40807 (10)	0.0226 (3)	
C8	0.44122 (8)	−0.2180 (3)	0.35634 (12)	0.0232 (3)	
C9	0.42870 (8)	−0.2295 (3)	0.24289 (12)	0.0227 (3)	
C10	0.38163 (7)	−0.0544 (2)	0.18193 (10)	0.0202 (2)	
C11	0.34623 (7)	0.1339 (2)	0.23375 (10)	0.0170 (2)	
C12	0.29775 (7)	0.3214 (2)	0.16423 (10)	0.0173 (2)	
H1	0.1213 (10)	−0.132 (3)	0.2126 (14)	0.028 (4)*	
H3	0.0695 (11)	0.375 (4)	−0.0377 (16)	0.040 (5)*	
H4	0.1539 (10)	0.154 (4)	−0.1271 (15)	0.032 (5)*	
H6	0.3370 (10)	0.270 (4)	0.3810 (14)	0.026 (4)*	
H7	0.4150 (10)	−0.018 (4)	0.4848 (14)	0.032 (5)*	
H8	0.4762 (10)	−0.344 (3)	0.4016 (14)	0.026 (4)*	
H9	0.4506 (10)	−0.362 (3)	0.2061 (14)	0.029 (4)*	
H10	0.3722 (9)	−0.058 (3)	0.1028 (13)	0.024 (4)*	
H1O2	0.2262 (13)	0.590 (5)	0.1647 (19)	0.061 (7)*	
H1N2	0.2563 (11)	−0.378 (4)	−0.0353 (16)	0.039 (5)*	
H2N2	0.2443 (11)	−0.186 (4)	−0.1250 (16)	0.033 (5)*	

### Atomic displacement parameters (Å^2^)

**Table:**

	*U*^11^	*U*^22^	*U*^33^	*U*^12^	*U*^13^	*U*^23^
Cl1	0.02624 (17)	0.02425 (18)	0.02760 (18)	0.00709 (12)	0.00407 (12)	0.00036 (12)
N1	0.0220 (5)	0.0181 (5)	0.0187 (5)	0.0018 (4)	0.0026 (4)	0.0026 (4)
N2	0.0345 (6)	0.0266 (6)	0.0217 (5)	0.0075 (5)	0.0096 (5)	0.0059 (5)
C1	0.0208 (5)	0.0187 (6)	0.0208 (6)	0.0005 (5)	0.0025 (4)	0.0021 (5)
C2	0.0185 (5)	0.0186 (6)	0.0228 (6)	0.0008 (5)	0.0011 (4)	−0.0009 (5)
C3	0.0232 (6)	0.0200 (6)	0.0240 (6)	0.0016 (5)	−0.0028 (5)	0.0040 (5)
C4	0.0249 (6)	0.0229 (6)	0.0193 (6)	0.0005 (5)	−0.0006 (5)	0.0049 (5)
C5	0.0208 (5)	0.0189 (6)	0.0193 (5)	−0.0018 (5)	0.0012 (4)	0.0010 (5)
O1	0.0320 (5)	0.0243 (5)	0.0148 (4)	0.0045 (4)	0.0059 (3)	0.0020 (4)
O2	0.0225 (4)	0.0211 (4)	0.0178 (4)	0.0043 (4)	0.0055 (3)	0.0031 (4)
C6	0.0207 (5)	0.0203 (6)	0.0182 (5)	0.0006 (5)	0.0063 (4)	0.0022 (5)
C7	0.0219 (6)	0.0267 (7)	0.0191 (6)	0.0010 (5)	0.0037 (4)	0.0056 (5)
C8	0.0197 (6)	0.0206 (6)	0.0291 (7)	0.0010 (5)	0.0034 (5)	0.0065 (5)
C9	0.0210 (6)	0.0182 (6)	0.0289 (7)	0.0010 (5)	0.0044 (5)	−0.0018 (5)
C10	0.0210 (5)	0.0195 (6)	0.0200 (5)	−0.0013 (5)	0.0033 (4)	−0.0026 (5)
C11	0.0165 (5)	0.0158 (5)	0.0187 (5)	−0.0029 (4)	0.0030 (4)	0.0015 (4)
C12	0.0179 (5)	0.0167 (6)	0.0172 (5)	−0.0025 (4)	0.0027 (4)	0.0001 (4)

### Geometric parameters (Å, °)

**Table:**

Cl1—C2	1.7382 (13)	O2—C12	1.3190 (15)
N1—C5	1.3454 (16)	O2—H1O2	0.98 (2)
N1—C1	1.3532 (17)	C6—C11	1.3936 (17)
N2—C5	1.3530 (18)	C6—C7	1.3946 (18)
N2—H1N2	0.88 (2)	C6—H6	0.907 (18)
N2—H2N2	0.881 (19)	C7—C8	1.392 (2)
C1—C2	1.3705 (18)	C7—H7	0.945 (17)
C1—H1	0.970 (18)	C8—C9	1.395 (2)
C2—C3	1.4006 (19)	C8—H8	1.015 (18)
C3—C4	1.368 (2)	C9—C10	1.3854 (19)
C3—H3	0.99 (2)	C9—H9	0.962 (18)
C4—C5	1.4149 (18)	C10—C11	1.4005 (18)
C4—H4	0.936 (18)	C10—H10	0.972 (16)
O1—C12	1.2250 (15)	C11—C12	1.4913 (17)
			
C5—N1—C1	118.92 (11)	C11—C6—H6	120.1 (11)
C5—N2—H1N2	120.0 (13)	C7—C6—H6	120.1 (11)
C5—N2—H2N2	119.3 (13)	C8—C7—C6	120.33 (12)
H1N2—N2—H2N2	117.8 (18)	C8—C7—H7	120.9 (11)
N1—C1—C2	122.21 (12)	C6—C7—H7	118.7 (11)
N1—C1—H1	118.2 (11)	C7—C8—C9	119.92 (12)
C2—C1—H1	119.5 (11)	C7—C8—H8	119.6 (10)
C1—C2—C3	119.61 (12)	C9—C8—H8	120.5 (10)
C1—C2—Cl1	120.10 (10)	C10—C9—C8	119.96 (12)
C3—C2—Cl1	120.25 (10)	C10—C9—H9	119.2 (11)
C4—C3—C2	118.61 (12)	C8—C9—H9	120.8 (11)
C4—C3—H3	118.7 (11)	C9—C10—C11	120.26 (12)
C2—C3—H3	122.6 (11)	C9—C10—H10	121.5 (10)
C3—C4—C5	119.45 (12)	C11—C10—H10	118.3 (10)
C3—C4—H4	121.2 (11)	C6—C11—C10	119.82 (12)
C5—C4—H4	119.3 (11)	C6—C11—C12	122.12 (11)
N1—C5—N2	117.98 (12)	C10—C11—C12	118.04 (11)
N1—C5—C4	121.20 (12)	O1—C12—O2	123.17 (11)
N2—C5—C4	120.81 (12)	O1—C12—C11	120.66 (11)
C12—O2—H1O2	111.7 (14)	O2—C12—C11	116.17 (10)
C11—C6—C7	119.69 (12)		
			
C5—N1—C1—C2	−0.16 (19)	C6—C7—C8—C9	0.6 (2)
N1—C1—C2—C3	−0.2 (2)	C7—C8—C9—C10	0.0 (2)
N1—C1—C2—Cl1	−178.03 (10)	C8—C9—C10—C11	−0.3 (2)
C1—C2—C3—C4	0.34 (19)	C7—C6—C11—C10	0.60 (19)
Cl1—C2—C3—C4	178.12 (10)	C7—C6—C11—C12	−177.84 (11)
C2—C3—C4—C5	−0.04 (19)	C9—C10—C11—C6	−0.01 (19)
C1—N1—C5—N2	−178.39 (12)	C9—C10—C11—C12	178.49 (11)
C1—N1—C5—C4	0.47 (19)	C6—C11—C12—O1	165.21 (12)
C3—C4—C5—N1	−0.37 (19)	C10—C11—C12—O1	−13.25 (18)
C3—C4—C5—N2	178.46 (13)	C6—C11—C12—O2	−14.87 (17)
C11—C6—C7—C8	−0.9 (2)	C10—C11—C12—O2	166.67 (11)

### Hydrogen-bond geometry (Å, °)

**Table:**

*D*—H···*A*	*D*—H	H···*A*	*D*···*A*	*D*—H···*A*
O2—H1O2···N1^i^	0.98 (2)	1.65 (2)	2.629 (1)	175 (2)
N2—H1N2···O1^ii^	0.88 (2)	2.04 (2)	2.898 (2)	165.4 (18)
N2—H2N2···O2^iii^	0.88 (2)	2.37 (2)	3.231 (2)	165.8 (17)
C3—H3···Cl1^iv^	0.99 (2)	2.82 (2)	3.780 (2)	163.4 (16)
C6—H6···O1^v^	0.91 (2)	2.58 (2)	3.095 (2)	116.3 (15)

Symmetry codes: (i) *x*, *y*+1, *z*; (ii) *x*, *y*−1, *z*; (iii) *x*, −*y*+1/2, *z*−1/2; (iv) −*x*, −*y*+1, −*z*; (v) *x*, −*y*+1/2, *z*+1/2.
